# NF-*κ*B inhibition reveals a novel role for HGF during skeletal muscle repair

**DOI:** 10.1038/cddis.2015.66

**Published:** 2015-04-23

**Authors:** J D Proto, Y Tang, A Lu, W C W Chen, E Stahl, M Poddar, S A Beckman, P D Robbins, L J Nidernhofer, K Imbrogno, T Hannigan, W M Mars, B Wang, J Huard

**Affiliations:** 1 Department of Orthopaedic Surgery, University of Pittsburgh School of Medicine, Pittsburgh, PA, USA; 2 Department of Bioengineering, University of Pittsburgh School of Medicine, Pittsburgh, PA, USA; 3 Department of Pathology, University of Pittsburgh School of Medicine, Pittsburgh, PA, USA; 4 Department of Metabolism and Aging, The Scripps Research Institute, Jupiter, FL

## Abstract

The transcription factor nuclear factor *κ*B (NF-*κ*B)/p65 is the master regulator of inflammation in Duchenne muscular dystrophy (DMD). Disease severity is reduced by NF-*κ*B inhibition in the *mdx* mouse, a murine DMD model; however, therapeutic targeting of NF-*κ*B remains problematic for patients because of its fundamental role in immunity. In this investigation, we found that the therapeutic effect of NF-*κ*B blockade requires hepatocyte growth factor (HGF) production by myogenic cells. We found that deleting one allele of the NF-*κ*B subunit p65 (*p65*^+/−^) improved the survival and enhanced the anti-inflammatory capacity of muscle-derived stem cells (MDSCs) following intramuscular transplantation. Factors secreted from *p65*^+/−^ MDSCs in cell cultures modulated macrophage cytokine expression in an HGF-receptor-dependent manner. Indeed, we found that following genetic or pharmacologic inhibition of basal NF-*κ*B/p65 activity, HGF gene transcription was induced in MDSCs. We investigated the role of HGF in anti-NF-*κ*B therapy *in vivo* using *mdx*;*p65*^+/−^ mice, and found that accelerated regeneration coincided with HGF upregulation in the skeletal muscle. This anti-NF-*κ*B-mediated dystrophic phenotype was reversed by blocking *de novo* HGF production by myogenic cells following disease onset. HGF silencing resulted in increased inflammation and extensive necrosis of the diaphragm muscle. Proteolytic processing of matrix-associated HGF is known to activate muscle stem cells at the earliest stages of repair, but our results indicate that the production of a second pool of HGF by myogenic cells, negatively regulated by NF-*κ*B/p65, is crucial for inflammation resolution and the completion of repair in dystrophic skeletal muscle. Our findings warrant further investigation into the potential of HGF mimetics for the treatment of DMD.

The transcription factor nuclear factor-*κ*B (NF-*κ*B) is highly activated in the skeletal muscle (henceforth simply termed ‘muscle') of patients suffering from Duchenne muscular dystrophy (DMD).^1^ In this fatal neuromuscular disease, absence of the cytoskeletal protein dystrophin results in muscle membrane instability, ongoing muscle degeneration and chronic inflammation. During early childhood, at the onset of disease, muscle is able to effectively repair. In early adolescence, however, tissue regeneration rapidly declines, and patients usually expire in their 20s.^1, 2^ It is well established from animal models of DMD, such as the dystrophin-deficient *mdx* mouse, that the dystrophic phenotype is exacerbated by NF-*κ*B activation. Genetic or pharmacologic strategies blocking p65, the classical NF-*κ*B DNA-binding subunit, or its upstream activator, I*κ*B kinase *β* (IKK*β*), have been found to reduce inflammation and accelerate muscle regeneration.^1^ This type of treatment approach is problematic for DMD patients, however, given the broad role of this transcription factor, particularly in immunity.^3^

NF-*κ*B suppression is thought to improve muscle regeneration by acting directly on immune cells and muscle progenitor cells to reduce inflammation and promote muscle differentiation, respectively.^4, 5^ We previously reported that NF-*κ*B negatively influences the myogenic potential of muscle-derived stem cells (MDSCs),^6^ a highly myogenic cell population with stem cell-like characteristics, including multilineage differentiation and self-renewal.^7, 8^ When compared with committed muscle precursor cells, or myoblasts, MDSCs demonstrate a higher intramuscular engraftment capability in both the skeletal and cardiac muscle.^9^ The improved regenerative potential of MDSCs may be related to stress resistance^10, 11^ and the release of soluble factors such as vascular endothelial growth factor (VEGF).^12, 13^ Of note, many secreted factors are NF-*κ*B target genes, suggesting that its blockade might have broader implications than promoting myogenic lineage progression. This is suggested by our earlier work, in which we unexpectedly observed that intramuscular injection of donor MDSCs haploinsufficient for *p65* reduced wild-type (WT) recipient muscle necrosis and CD14+ cell infiltration significantly more than WT MDSCs.^6^ This suggests that enhanced differentiation in *mdx* skeletal muscle may not account for all of the benefit of NF-*κ*B blockade, and perhaps may offer a new strategy to reduce inflammation without compromising host immunity.

We found that loss of one *p65* allele improved the anti-inflammatory properties of MDSCs in a macrophage hepatocyte growth factor (HGF) receptor-, Met, dependent manner. In muscle, the processing of HGF sequestered in the extracellular matrix is responsible for the activation and migration of satellite cells following injury.^14, 15^ However, an active role for HGF/Met in the remaining stages of muscle regeneration and inflammation resolution has yet to be identified.^16, 17^ Herein, we investigated the importance of HGF in mediating the beneficial effect of NF-*κ*B/p65 blockade in dystrophic muscle using *mdx*;*p65*^+/−^ mice, a previously described genetic model of NF-*κ*B blockade in DMD.^1^ We found that enhanced muscle regeneration in *mdx*;*p65*^+/−^ mice coincided with significant HGF upregulation. To examine the role of HGF produced by muscle cells during repair, we blocked *de novo* HGF expression following the onset of the *mdx* pathology using a musculotropic adeno-associated viral (AAV) vector carrying HGF shRNA. HGF silencing resulted in significantly increased inflammation and necrosis in the *mdx*;*p65*^+/−^ diaphragm (DIA), thus reversing the beneficial effect of *p65* haploinsufficiency. Our findings identify a critical role for HGF during inflammation resolution and provide new mechanistic insight into how modulation of NF-*κ*B/p65 attenuates muscular dystrophy. With HGF mimetics currently in phase II clinical trials for treating myocardial infarction and kidney dysfunction, our results suggest that these drugs might also be used to control chronic inflammation in muscular dystrophy and other inflammatory myopathies without compromising host immunity.

## Results

### *P65* haploinsufficiency enhances MDSC anti-inflammatory properties

**Figure 1:**
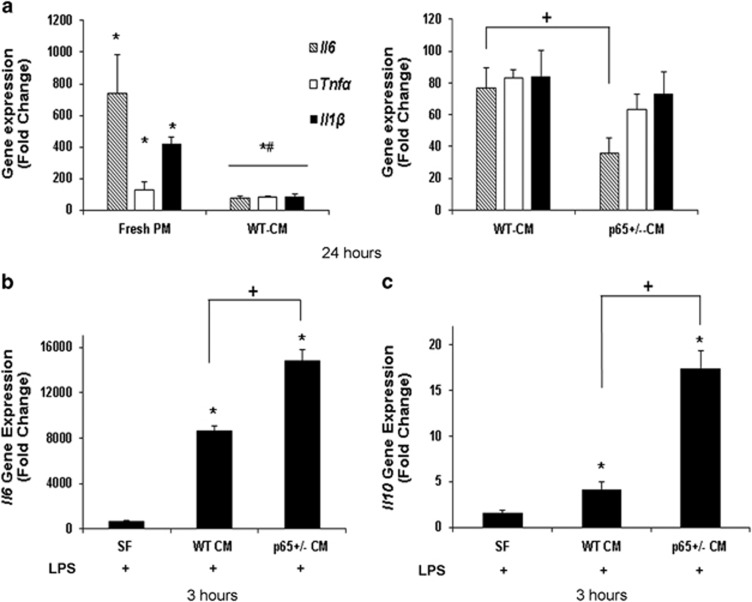
MDSC-CM reduces cytokine expression by LPS-activated RAW cells. (**a**) Real-time RT-PCR demonstrated that at 24 h, *Il1β*, *Tnfα* and *Il6* gene expression was attenuated by WT-CM (left), but the expression of *Il6* was even further reduced in *p65*^+/−^*-*CM-treated RAW cells (right) (*versus No LPS, *P*⩽0.05; ^#^*versus* +LPS, *P*⩽0.05; ^+^*versus* LPS+WT-CM, *P*⩽0.05). At an earlier timepoint of 3 h, (**b**) *Il6* and (**c**) *Il10* gene expression is induced in RAW cells by exposure to LPS and MDSC-CM, an effect that is enhanced in *p65*^+/−^ MDSCs (*versus LPS in SF medium, *P*⩽0.05; ^+^*versus* LPS+WT-CM, *P*⩽0.05). Data are displayed from a representative experiment as mean±S.E.M. Each experiment was performed a minimum of three times

We previously reported that compared with WT cells, intramuscular engraftments of donor *p65*^+/−^ MDSCs resulted in significantly reduced necrosis and inflammation in recipient tissue.^6^ To explore the mechanism behind this finding *in vitro*, we compared the ability of WT- and *p65*^+/−^ MDSC-conditioned medium (CM) to modulate the expression of prototypical cytokines in a murine macrophage cell line (RAW264.7; RAW). Briefly, RAW cells were exposed to lipopolysaccharide (LPS) in WT- or *p65*^+/−^-CM for 24 h. Exposure to WT- or *p65*^+/−^-CM was associated with reduced tumor necrosis factor-*α* (*Tnfα*), interleukin-1*β* (*Il1β*) and interleukin-6 (*Il6*) transcription (Figure 1a, left). Interestingly, we found that it was the RAW cells stimulated in *p65*^+/−^-CM that demonstrated the greatest suppression of *Il6* (a 10-fold decrease in *p65*^+/−^-CM *versus* WT-CM, *P*<0.05) (Figure 1a). As *Il6* is a secondary response gene, we suspected that earlier events in the cytokine cascade were modulated by MDSC-secreted factors. Therefore, we continued these experiments at an earlier time point and under serum-free (SF) conditions. We found that at 3 h, RAW cells stimulated with LPS in *p65*^+/−^-CM demonstrated a significant induction of *Il6*, in contrast to the decrease we had observed at 24 h. Additionally, we observed induction of the anti-inflammatory factor interleukin-10 (*Il10)* (Figures 1b and c; *P*<0.05). WT-CM had a similar effect, but to a significantly smaller degree (Figures 1b and c; *P*<0.05). In contrast, LPS alone in the fresh medium did not induce *Il10* at 3 h (*P*=0.11). Of note, the accelerated induction of both *Il6* and *Il10* may explain the decrease in *Il6* we observed at 24 h. These results demonstrate that immunomodulatory factors regulated by NF-*κ*B/p65 are secreted by MDSCs under basal conditions.

### *P65*^ +/−^ MDSC engraftments accelerate healing in injured muscle when compared with WT MDSC engraftments

**Figure 2:**
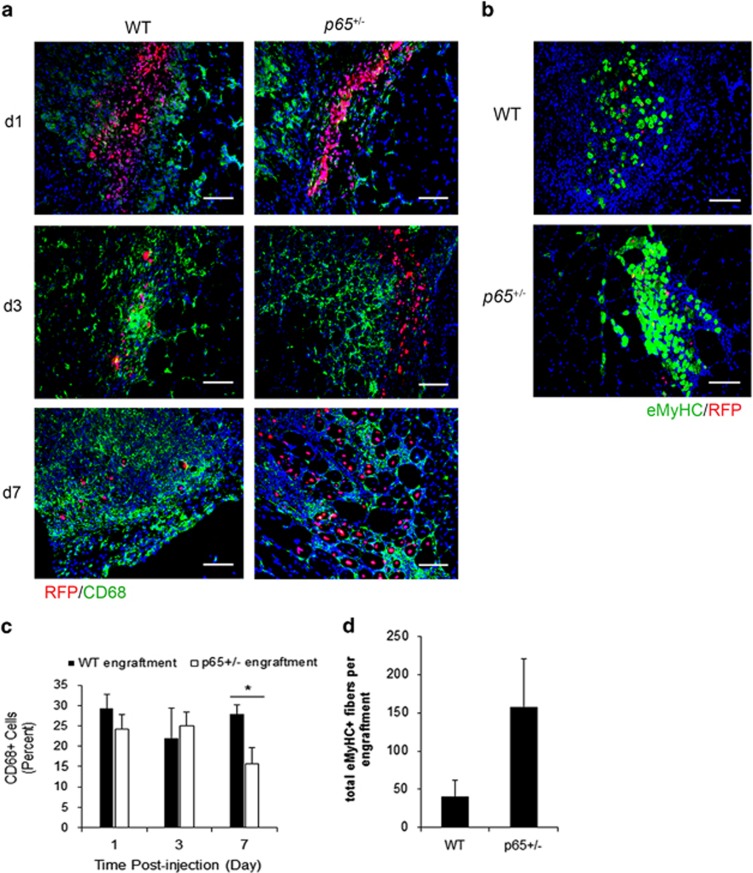
Donor *p65*^+/−^ MDSC engraftments promote the repair of recipient muscle. (**a**) Immunofluorescent staining of tissue for the macrophage marker CD68 (green) indicated that RFP+ donor cell (red) engraftments in injured muscle were infiltrated by macrophages within 24 h postinjection (48 h postinjury), which continued to persist at 7 days (bottom). (**b**) eMyHC+ fibers (green) could be identified in or around donor cell engraftments at 7 days. (**c**) Quantification of CD68 positivity within x20 images indicated that *p65*^+/−^ MDSC engraftments have significantly less CD68+ cells present at 7 days (*P⩽0.05), and (**d**) demonstrated a trend towards higher numbers of total eMyHC+ fibers (host+donor) (*P*=0.12). Data are displayed as mean±S.E.M.; *n*=3–4 mice per group. Scale bar: 100 *μ*m

Although we previously reported that reduced numbers of CD14+ cells were associated with *p65*^+/−^ MDSC engraftments at 1 week postinjection,^6^ a time-course analysis was required to determine if this finding was reflective of accelerated inflammation resolution. Thus, we used a similar cell transplant model to monitor recipient inflammation up to 7 days following intramuscular injection of WT or *p65*^+/−^ MDSCs. Briefly, the gastrocnemius (GAS) muscle was injured with cardiotoxin (CTX), and 24 h later, MDSCs retrovirally labeled with nuclear-localized red fluorescent protein (RFP) were delivered intramuscularly. Immunofluorescent staining of tissue sections revealed that *p65*^+/−^ cell engraftments were associated with significantly less CD68+ cells at day 7, but not at days 1 and 3 (Figures 2a and c). Nearly all CD68+ cells coexpressed CD11b (data not shown); therefore, CD68 was used as a macrophage marker in subsequent experiments. Staining for the embryonic isoform of myosin heavy chain (eMyHC) identified regenerating fibers local to donor cell engraftments at day 7 (Figure 2b). *p65*^+/−^ MDSC engraftments demonstrated a trend (*P*=0.12) towards being associated with more eMyHC+ myofibers (Figure 2d). This demonstrates that inflammation resolution, likely coupled with regeneration, was accelerated by *p65*^+/−^ MDSC engraftment.

### Improved survival of donor *p65*^ +/−^ MDSCs in injured WT muscle at 3 and 7 days postinjection

**Figure 3:**
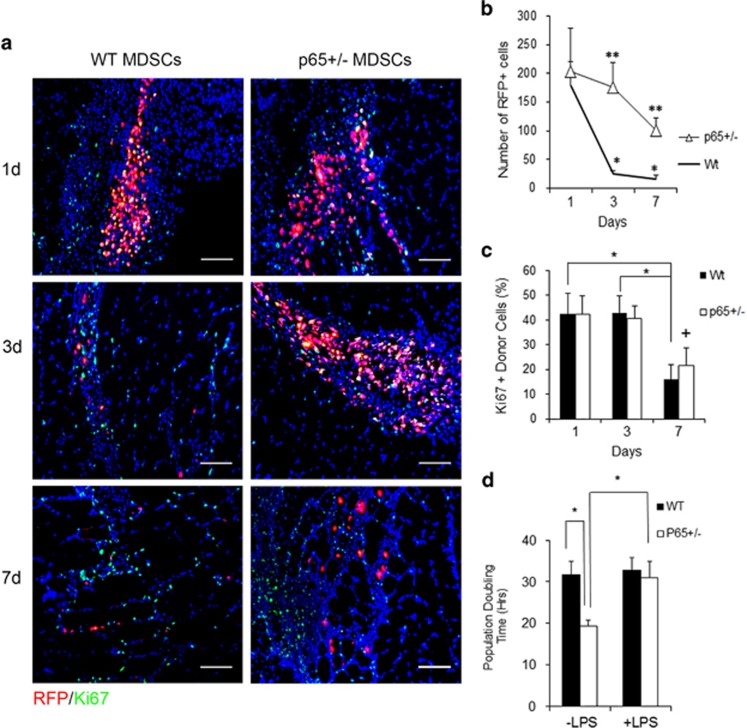
Haploinsufficiency of *p65* improves MDSC survival *in vivo*. (**a**) Immunofluorescent staining of tissue sections for the proliferation marker RFP (donor cells, red) and Ki-67 (green) demonstrated improved survival of *p65*^+/−^ MDSCs up to 1 week postinjection. (**b**) Quantification of RFP+ cells indicated a significant decline in WT cells within the first week, while *p65*^+/−^ MDSCs displayed a much slower decline in number (**versus WT, *P*⩽0.001; *versus day 1, *P*⩽0.05). (**c**) Ki-67 positivity indicated that there were no differences in proliferation at days 1 and 3 (*P⩽0.05; ^+^*versus* day 1, *P*⩽0.10). (**d**) When cocultured with RAW cells (1 : 10) in the presence of LPS, the population doubling time of *p65*^+/−^ MDSCs significantly increased, reflecting a decreased rate of proliferation. WT MDSCs demonstrated no significant changes (*P<0.05). For (**a**): Scale bar: 100 *μ*m; *n*=8–9 mice per group. Data are displayed as mean±S.E.M. Data in (**d**) are from one experiment representative of three

As evident in Figure 2, and confirming our previous investigation, engraftments of *p65*^+/−^ MDSCs appeared larger than those of WT cells.^6^ Indeed, the number of WT cells declined rapidly from days 1 to 3 (Figures 3a and b; *P*<0.05). It is unlikely that the higher number of *p65*^+/−^ cells was because of proliferation, as staining for Ki-67 indicated no difference between groups (Figures 3a and c). This was surprising because *in vitro*, *p65*^+/−^ MDSCs demonstrate higher proliferation than WT cells.^6^ To further determine how inflammation might affect the expansion of the donor MDSCs, we cocultured MDSCs with RAW cells *in vitro* (ratio of 1 : 10). We determined MDSC population doubling time (PDT) using a previously validated model of cell population growth.^18^ In the absence of LPS, *p65*^+/−^ MDSCs were more proliferative compared with WT cells (Figure 3d).^6^ However, the addition of LPS extended the PDT of *p65*^+/−^ cells, but not WT cells, indicating a decrease in *p65*^+/−^ cell proliferation. Thus, decreased proliferation, in addition to paracrine modulation of the inflammatory microenvironment, may represent a mechanism through which *p65*^+/−^ MDSCs are protected from cell death following transplantation to injured muscle.

### Inhibition of IKK*β* in MDSCs induces *Hgf* expression

**Figure 4:**
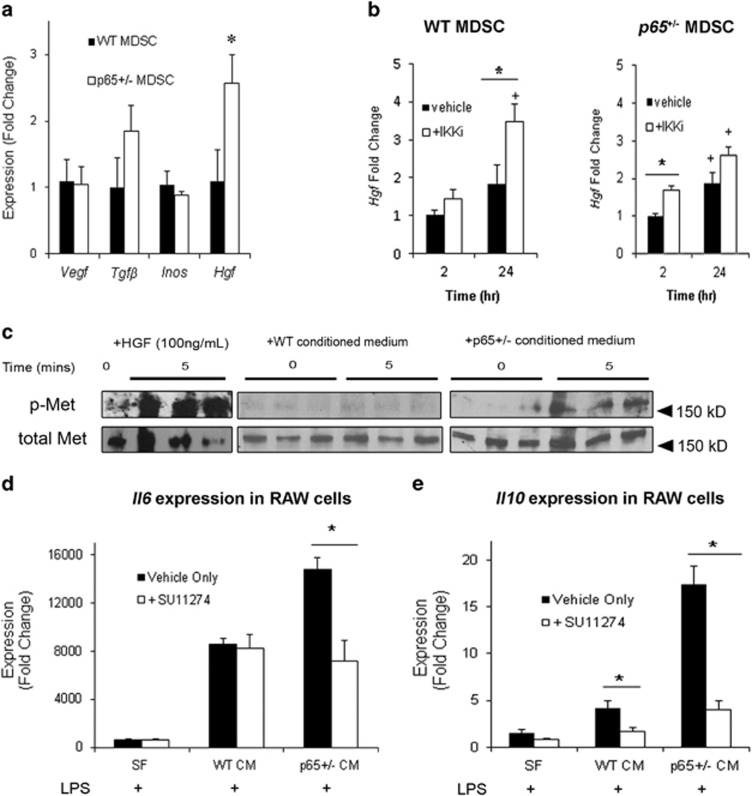
Reducing basal NF-*κ*B activity upregulates *Hgf* expression in MDSCs. (**a**) Real-time RT-PCR analysis revealed that *Hgf* was significantly upregulated in *p65*^+/−^ compared with WT MDSCs (*P<0.05). (**b**) *Hgf* transcription could also be induced in WT cells by treatment with an IKKi for 24 h (left). IKKi treatment of *p65*^+/−^ MDSCs induced only a modest increase in *Hgf* at 2 h (*versus vehicle, *P*<0.05; ^+^*versus* time=2 h; *P*<0.05). (**c**) p-Met in RAW cells can be detected within 5 min of stimulation with HGF (left), or *p65*^+/−^ CM (right), but not WT-CM (middle). (**d**) Inhibiton of Met activation on RAW cells using SU11274 significantly decreased *Il6* induction 3 h following stimulation with LPS and *p65*^+/−^-CM, but not LPS and WT-CM. (**e**) *Il10* induction by LPS in both WT-CM or *p65*^+/−^ CM was decreased by SU11274, but to a much greater degree in the *p65*^+/−^ CM group (*versus vehicle, *P*<0.05). Values from a representative experiment are displayed as mean±S.E.M. Each experiment was performed at least three times

We next endeavored to identify anti-inflammatory factors differentially expressed by *p65*^+/−^ MDSCs. We began with a number of candidate genes known to have immunomodulatory properties, including transforming growth factor *β*1 (*Tgfb*), *Vegf*, *Hgf*, interleukin-4 (*Il4*), *Il10* and inducible nitric oxide synthase (*Inos*).^19^ Of these factors, only *Hgf* was significantly upregulated (Figure 4a, *P*⩽0.05). *Il4* and *Il10* transcripts were detected in neither WT nor *p65*^+/−^ MDSCs (data not shown). Under basal conditions, proliferating myogenic cells display a relatively high level of NF-*κ*B activity.^20^ To verify a relationship between *Hgf* expression and NF-*κ*B activity, we treated WT and *p65*^+/−^ cells with an inhibitor of IKK*β* (IKK-2 inhibitor IV, IKKi) and examined gene expression after 2 and 24 h. By 24 h, *Hgf* was upregulated ~+3.5-fold (*P*⩽0.05) in IKKi-treated WT MDSCs (Figure 4b). *p65*^+/−^ MDSCs demonstrated a small increase (+1.67-fold, *P*⩽0.05) after 2 h, but at 24 h this did not significantly differ from the vehicle alone (*P*=0.46). These experiments confirm a negative relationship between *Hgf* and IKK*β*/NF-*κ*B in MDSCs.

### *P65*^*+*/−^-CM modulation of *Il10* and *Il6* expression in RAW cells depends on the HGF receptor, Met

To verify the functional significance of increased HGF transcription, we examined the activation of its receptor, Met, in RAW cells exposed to CM. Strikingly, phosphorylated Met (p-Met) was detectable at 5 min following exposure to *p65*^+/−^-CM, but not WT-CM (Figure 4c, right, middle). Stimulating cells with LPS and CM in the presence of the Met inhibitor, SU11274 (Sigma, St. Louis, MO, USA), significantly decreased the induction of *Il6* and *Il10* by *p65*^+/−^ CM observed after 3 h (Figures 4d, *P*⩽0.05), demonstrating that MDSC modulation of RAW cell gene transcription occurs downstream of Met. *Il6* induction by WT-CM was not significantly changed. *Il10* induction did decrease, although to a lesser degree than that of the *p65*^+/−^ CM group (Figures 4e, *P*<0.05).

**Figure 5:**
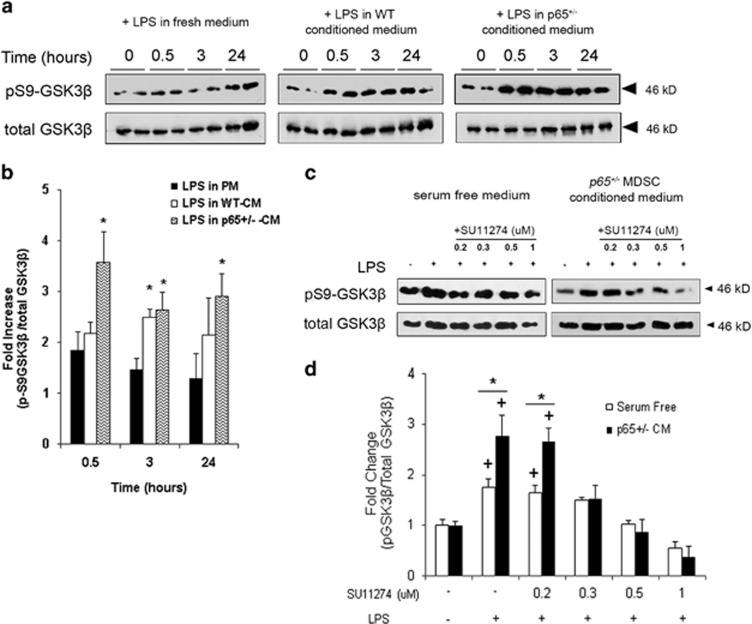
*p65*^+/−^-CM activates an HGF/Met/GSK3*β* pathway in RAW cells. (**a**) Western blot demonstrated that activation of RAW cells in PM induced an increase in pS9-GSK3*β* within 30 min (left), a response that was amplified by both WT- (middle) and *p65*^+/−^*-*CM (right). (**b**) Densitometric analysis revealed that when activated in *p65*^+/−^-CM, the fraction of pS9-GSK3*β* increased by 3.5-fold in 30 min, an amount significantly higher than WT-CM and PM groups (*P⩽0.05). (**c**) Inhibition of Met by SU11274 blocked pS9-GSK3*β* in RAW cells 30 min after exposure to LPS and *p65*^+/−^-CM (**d**) in a dose-dependent manner (*versus SF+LPS, ^+^*versus* no LPS, *P*<0.05) Data are represented as mean±S.E.M. of at least three independent experiments

HGF/Met has been reported to modulate cytokine production by the inactivation of glycogen synthase kinase 3*β* (GSK3*β*).^21^ Under basal conditions, GSK3*β* sequesters transcription factors that are required for the induction of anti-inflammatory genes. For example, *Il10* is induced following inactivation of GSK3*β* via phosphorylation on serine 9 (pS9-GSK3*β*).^22^ By western blot, we found that at 30 min, RAW cells activated in *p65*^+/−^*-*CM demonstrated a striking +3.5-fold (*P*⩽0.05) increase in pS9-GSK3*β*, which remained elevated (+2.9-fold, *P*⩽0.05) up to 24 h. In contrast, when activated in WT-CM or fresh medium, pS9-GSK3*β* increased to a significantly smaller degree and then declined (Figures 5a and b). Moreover, GSK3*β* phosphorylation 30 min postexposure to LPS in *p65*^+/−^-CM was abrogated by treatment with SU11274 (Sigma) (Figure 5c and d). Collectively, these results suggest that HGF released from *p65*^+/−^ MDSCs modulates cytokine gene expression in activated macrophages via inactivation of macrophage GSK3*β*.

### Accelerated regeneration in *p65*^ +/−^ skeletal muscle is accompanied by elevated HGF and macrophage pS9-GSK3*β*

**Figure 6:**
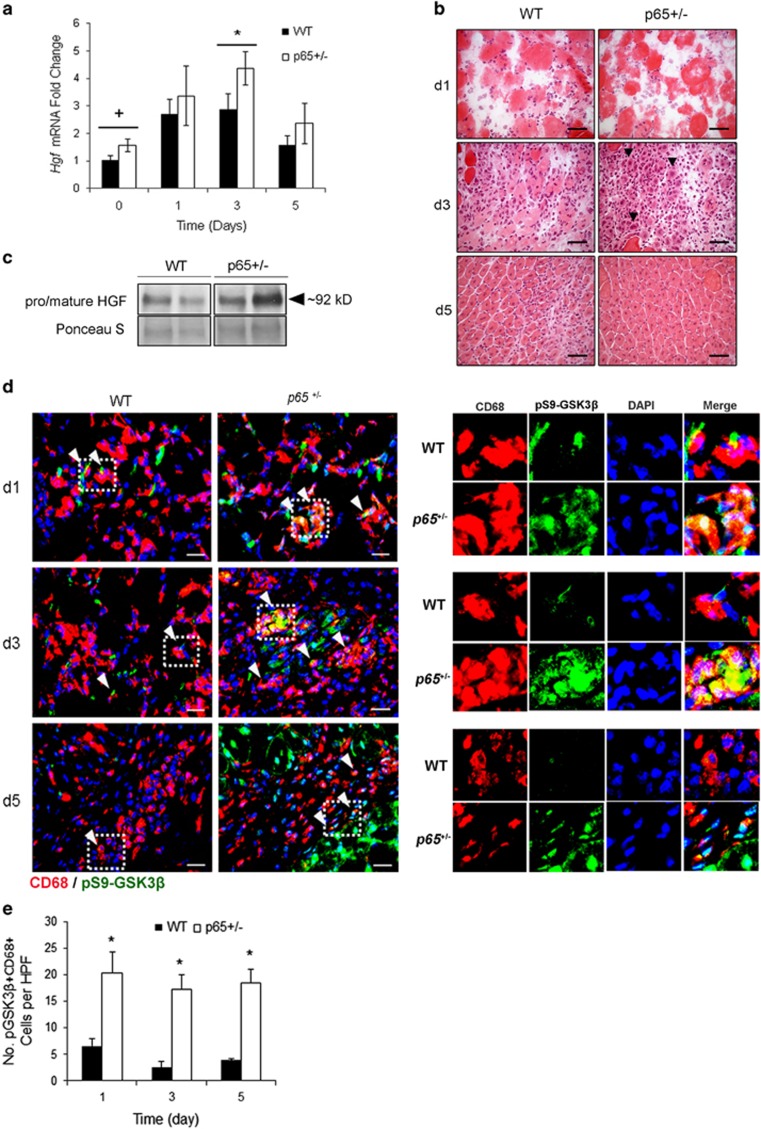
*Hgf* upregulation correlates with accelerated muscle regeneration *in vivo*. (**a**) *Hgf* expression was significantly upregulated in *p65*^+/−^ muscle at 3 days after CTX injury (*P⩽0.05 *versus* 3 days WT; ^+^*P*<0.10 *versus* day 0 WT). (**b**) Representative images from hemotoxylin and eosin staining indicated that compared with WT muscle, the GAS muscle of *p65*^+/−^ mice regenerated more rapidly (arrows) following CTX injury. (**c**) Non-reducing sodium dodecyl sulfate-polyacrylamide gel electrophoresis (SDS-PAGE) and western blot analysis of muscle extracts showed a slight increase in total HGF in uninjured *p65*^+/−^ muscles. (**d**) pS9-GSK3*β*+ macrophages were identified in injured skeletal muscle by immunofluorescent costaining for pS9-GSK3*β* (green) and CD68 (red). Arrows indicate examples of colocalization and regions outlined with dashes are digitally enlarged and separated by color channel to the right. (**e**) Quantification of the number of pS9-GSK3*β*+ macrophages per high power field (HPF, x600) indicated that a significantly higher number of pS9-GSK3*β*^+^/CD68^+^ macrophages were found in *p65*^+/^^−^ skeletal muscle compared with WT skeletal muscle at 1, 3 and 5 days after injury (*P*⩽0.05). For (**b**), scale bar: 50 *μ*m, *n*=6–8 mice per group. For (**d**), scale bar: 20 *μ*m, *n*=3 mice per group. Data are displayed as mean±S.E.M.

To determine whether the macrophage HGF/Met/GSK3*β* pathway is activated during muscle regeneration *in vivo*, we conducted CTX injury experiments on 4- to 5-week-old *p65*^+/−^ and WT mice. In uninjured *p65*^+/−^ muscles, higher levels of *Hgf* mRNA were detected but this was not statistically significant (Figures 6a, *P*=0.09). Similarly, uninjured *p65*^+/−^ muscle had a modest increase in total HGF protein (Figure 6c). We found *Hgf* expression was significantly higher in *p65*^+/−^ muscle after 3 days (Figure 6a). This coincided with *de novo* fiber formation, visible by H&E staining of tissue sections (Figure 6b, middle panel, arrows). Consistent with the findings of Archaryya and co-workers^1^ regarding accelerated muscle regeneration in *p65*^+/−^ mice, muscle inflammation was reduced at 5 days postinjury (Figure 6b). Immunohistochemical staining revealed that compared with WT muscle, HGF-positive mononuclear cells occupied a greater area in *p65*^+/−^ muscle at 1 and 3 days postinjury (Supplementary Figure S1A). We next isolated macrophage and myoblast populations from WT and *p65*^+/−^ hindlimb muscles at day 3 postinjury. Not only were significantly less macrophages identified as F4/80+ cells, collected from *p65*^+/−^ muscle (Supplementary Figure S1B), but also *ex vivo* analysis demonstrated no difference in *Hgf* expression and only a small, nonsignificant difference in HGF secretion (Supplementary Figure S1C). In contrast, TNF*α* stimulation of primary *p65*^+/−^ myoblasts resulted in stronger induction of *Hgf* expression compared with WT myoblasts (Supplementary Figure S1D). Collectively, these data indicate that myogenic progenitor cells are the likely source of elevated *Hgf* in CTX-injured *p65*^+/−^ muscle.

We next stained tissue sections for CD68 and pS9-GSK3*β* (Figure 6d). Compared with WT muscle, a substantially higher number of pS9-GSK3*β* macrophages was found in *p65*^+/−^ muscle at all three time points tested (Figure 6e, *P*⩽0.05). *Ex vivo* analysis of macrophages isolated on day 3 postinjury revealed that *p65*^+/−^ macrophages expressed higher levels of both *Il6* and *Il10* compared with WT cells (Supplementary Figure S2A), consistent with our *in vitro* results using RAW cells treated with *p65*^+/−^ MDSC-CM (Figure 4). Higher secretion of IL-10 by *p65*^+/−^ macrophages was further confirmed by ELISA (Supplementary Figure S2B). Of note, upon stimulation with an equal amount of HGF (±LPS), WT and *p65*^+/−^-resident peritoneal macrophages did not demonstrate any differences in the percentage of pS9-GSK3*β*-positive cells *in vitro* (Supplementary Figure S2C). These results indicate that *p65* deficiency had no confounding effect on the HGF/Met/GSK3*β* pathway in native macrophages. Additionally, macrophage populations isolated from *p65*^+/−^ muscles contained higher percentages of cells expressing resistin-like molecule-*α* (RELM*α*) or CD163, markers of a prohealing, regenerative phenotype (Supplementary Figure S2D).^23, 24^ Taken together, these results demonstrate that the accelerated regeneration of *p65*^+/−^ muscle correlates with HGF upregulation in myogenic progenitor cells, leading to the modulation of macrophage phenotype, likely via a Met/GSK3*β* pathway.

### HGF is upregulated in *mdx;p65*^ +/−^ skeletal muscle during regeneration

**Figure 7:**
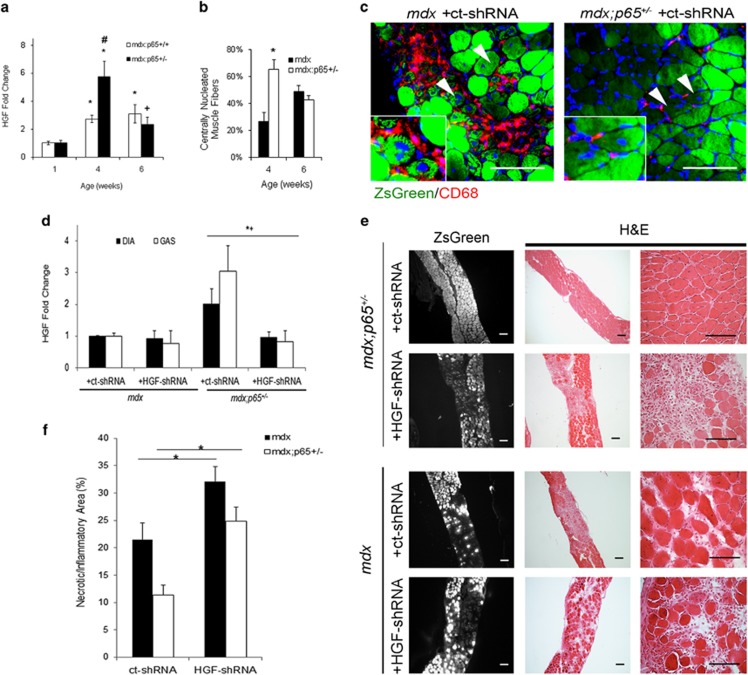
Upregulation of *Hgf* in *mdx;p65*^+/−^ skeletal muscle at 4 weeks of age correlates with accelerated regeneration. (**a**) Real-time RT-PCR demonstrated that *Hgf* expression was elevated in *mdx:p65*^+/−^ muscle at 4 weeks, coinciding with enhanced regeneration, quantified as the percent of centrally nucleated muscle fibers in (**b**) (*versus WT, *P*⩽0.05; ^+^*versus* WT, *P*⩽0.10; ^#^*versus mdx*, *P*⩽0.05). (**c**) ZsGreen+ centrally nucleated fibers indicated that muscle progenitor cells were transduced by the AAV vector (arrows, top). (**d**) After 4 weeks, *Hgf* expression was significantly reduced in the DIA and GAS muscles of HGF-shRNA-treated *mdx;p65*^+/−^ mice compared with the ct-shRNA-treated group (*P*⩽0.05, ^+^shHGF *versus* ct-shRNA; *GAS, ^+^DIA). (**e**) Silencing of *Hgf* worsens the histopathology of the DIA muscle from treated *mdx;p65*^+/−^ mice (top) and *mdx* mice. (**f**) We quantified the necrotic/inflammatory lesions in H&E-stained DIA tissue sections from treated mice by measuring the lesions as percent area; *n*=4–6 mice per group. Data are displayed as mean±S.E.M.

Based on our findings with the CTX injury model, we hypothesized that elevation of HGF contributes to the beneficial effects of NF-*κ*B blockade in dystrophic muscle. To investigate this, we used *mdx;p65*^+/−^ mice, a genetic model of NF-*κ*B/p65 inhibition, which has been reported to have a significantly attenuated dystrophic phenotype.^1^ We examined HGF expression in the GAS muscles of *mdx*;*p65*^+/−^ and *mdx;p65*^*+/+*^(*mdx*) littermates during both the degenerative (~4 weeks) and regenerative phases (~6 weeks) of muscle pathology. Strikingly, HGF was upregulated +5.7-fold in *mdx;p65*^+/−^ mice at 4 weeks of age compared with WT mice (Figures 7a, *P*<0.05). In contrast, HGF expression in *mdx* GAS only increased +2.7-fold. At this time, H&E staining of *mdx*:*p65*^+/−^ GAS revealed a reduced mononuclear cell infiltrate and significantly enhanced regeneration compared with *mdx* littermates (Figures 7b (*P*<0.05) and Supplementary Figure S3). By 6 weeks, we detected no significant differences in *Hgf* expression or regenerating fibers between *mdx* and *mdx*;*p65*^+/−^ GAS (Figures 7a and b). Importantly, we found no differences in *Hgf* expression in muscle tissues of presymptomatic, 1-week-old mice (Figure 7a). This demonstrated that upregulation of *Hgf* occurs following the onset of muscle degeneration and was associated with the regenerative stage of the *mdx* pathology, which was accelerated by ~2 weeks in *mdx;p65*^+/−^ mice.

### *In vivo* silencing of skeletal muscle-derived HGF reversed the ameliorated phenotype of *mdx;p65*^ +/−^ mice

We hypothesized that HGF upregulation precedes and subsequently promotes muscle regeneration. To investigate this, we designed a musculotropic recombinant AAV vector^25^ carrying the ZsGreen reporter gene and shRNA targeting HGF (HGF-shRNA). Five-day-old *mdx*;*p65*^+/−^ and *mdx* littermates received intraperitoneal injections of HGF-shRNA or control vector with scrambled shRNA (ct-shRNA). With this approach, the matrix reservoir of pro-HGF produced during development will be present until muscle degeneration begins, at ~3 weeks of age.^15^ Muscle-derived HGF produced during repair, however, will be targeted by our vector. Based on reporter gene expression, we observed high gene transfer efficiency in the limb and DIA muscles (Supplementary Figure S4A). Regenerating fibers exhibited ZsGreen expression, indicating that muscle progenitor cells were successfully transduced (Figure 7c, arrows). CD68 and ZsGreen did not colocalize, demonstrating that myeloid cells (red) were not transduced (Figure 7c). Finally, transduction of the liver and spleen was minimal, attesting to the musculotropic nature of our vector (Supplementary Figure S4B). These observations indicate that we preferentially transduced muscle fibers and progenitor cells using this approach.

**Figure 8:**
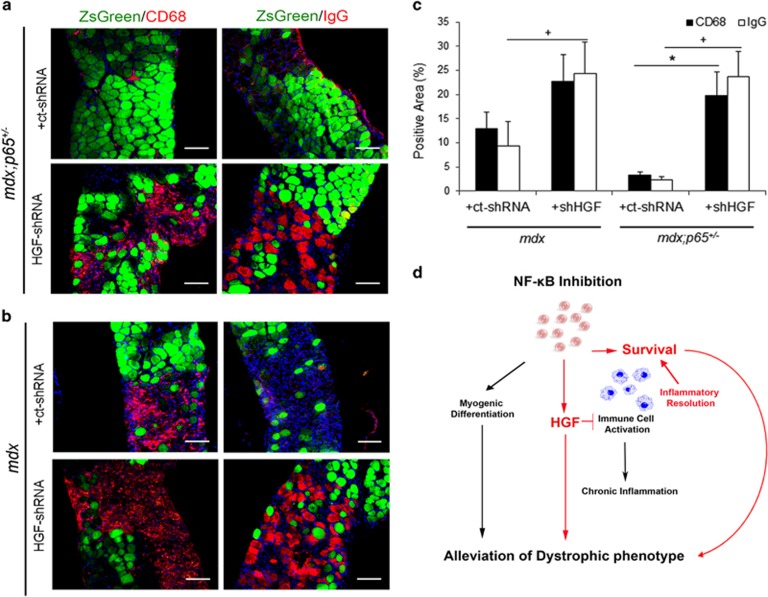
(**a**) Immunofluorescent staining revealed a striking increase in CD68+ macrophages (left) and IgG+ necrotic fibers (right) in the DIA of *mdx;p65*^+/−^-treated with HGF-shRNA compared with ct-shRNA *mdx;p65*^+/−^. (**b**) The DIA of HGF-shRNA-treated *mdx* mice did not demonstrate a statistically significant increase in inflammation (left), but did demonstrate a significant increase in fiber necrosis (right), quantified in (**c**) (*P⩽0.05 *versus* CD68 in ct-shRNA group; ^+^*P*⩽0.05 *versus* IgG in ct-shRNA group). (**d**) We propose a model in which NF-*κ*B inhibition improves dystrophic muscle regeneration not only by directly promoting lineage progression of muscle progenitor cells but also by increasing progenitor cell survival and upregulating the expression of *Hgf*. In turn, HGF may promote muscle fiber survival and inactivate myeloid cells to alleviate chronic inflammation in dystrophic muscle and promote repair. Scale bar: 100 *μ*m; *n*=4–6 mice per group. Data are displayed as mean±S.E.M.

HGF-shRNA reduced *Hgf* transcript by ~−2- and −4-fold in *mdx;p65*^+/−^ DIA and GAS, respectively (*P*⩽0.05) (Figure 7d). Paradoxically, *Hgf* in *mdx* DIA or GAS muscles was not significantly decreased by HGF-shRNA (*P*=0.80 and *P*=0.20, respectively). This may be because of a low level of *Hgf* in *mdx* muscles, relative to those of *mdx*;*p65*^+/−^ mice. HGF-shRNA-treated muscle demonstrated striking morphological changes compared with ct-shRNA-treated muscle. Transduction was highest in the DIA (Supplementary Figure S4A, data not shown); therefore, we focused on this muscle for our subsequent analysis. Increased degeneration of the DIA indicated a reversal of the histological improvements associated with *p65* haploinsufficiency, as shown in Figure 7e (top). Although the decrease in *Hgf* was not statistically significant in the *mdx* group, we still detected histological changes in treated muscle (Figures 7e and f). HGF-shRNA significantly increased the necrotic/inflammatory lesion area of *mdx*;*p65*^+/−^ DIA, such that the size of lesions (percent area) resembled those of *mdx* mice (Figure 7f). Similarly, the necrotic/inflammatory lesion area of *mdx* DIA increased following HGF-shRNA treatment. To distinguish between necrosis and inflammation, we identified necrotic fibers and infiltrating macrophages by mouse IgG and CD68 immunostaining, respectively. HGF silencing significantly increased both inflammatory and necrotic lesions of *mdx*;*p65*^+/−^ DIA (*P*⩽0.05) (Figures 8a and c). In contrast, only the IgG+ area was significantly increased in HGF-shRNA-treated *mdx* mice (*P*⩽0.05) (Figure 8c). Based on our results, HGF produced by muscle cells during repair is essential for the completion of regeneration. More importantly, these results indicate that HGF is critical for the beneficial effects of NF-*κ*B/p65 blockade on dystrophic muscle.

## Discussion

Numerous studies suggest that chronic inflammation accounts for a significant portion of muscle damage in DMD. The transcription factor NF-*κ*B is central to inflammation and has been found to be dysregulated in muscular dystrophy.^26^ In an earlier investigation by Archaryya and co-workers,^1^ the phenotype of *mdx* mice was found to be significantly attenuated by deletion of one *p65* allele. By conditional knockout of IKK*β* in either myeloid cells or muscle, they attributed this improvement to reduced inflammation and enhanced myogenesis, respectively. Indeed, p65 potently suppresses myogenic progenitor cell differentiation.^1, 4, 5, 6^ In this study, we further investigated effector mechanisms of anti-NF-*κ*B therapy and found that the alleviation of muscular dystrophy in *mdx*;*p65*^+/−^ mice depends in large part on HGF. Based on our results and summarized in Figure 8d, we propose that in addition to promoting myogenesis, NF-*κ*B blockade increases HGF expression by myogenic cells during repair, which acts locally to modulate inflammation and increase cell survival.

A regenerative role for HGF has been reported in a number of diverse adult tissues, such as liver, kidney and bone.^27, 28, 29^ Secreted in a proform, HGF requires cleavage by proteases to produce two chains, which bind to form the active HGF heterodimer.^30, 31^ In muscle, pro-HGF is stored in the extracellular matrix and is crucial for the activation of satellite cells following injury.^16, 32^ Unlike the models put forth by others, our work suggests that HGF is also important for the completion of regeneration. We found significantly higher *Hgf* expression in *mdx;p65*^+/−^ muscle at 4 weeks of age, correlating with reduced leukocyte infiltration and increased muscle fiber formation. The phenotypic improvements of *mdx;p65*^+/−^ muscle were reversed by silencing HGF preferentially in myogenic cells, resulting in significant degeneration of the DIA. Thus, stimulation of the HGF/Met pathway may be a viable strategy for treating inflammatory myopathies, such as DMD.

Notably, *p65* haploinsufficiency improved the survival and engraftment of donor MDSCs in host muscle. We have previously reported that the engraftment of myoblasts was improved by retroviral gene transfer of the anti-inflammatory mediator IL-1 receptor antagonist.^33^ Thus, it is reasonable to suggest that the local anti-inflammatory effects of HGF might be responsible for improved survival of donor cells. Further experiments will be required to investigate this point. The anti-inflammatory effect of HGF/Met in target cells appears to be mediated through inactivation of the ubiquitously expressed kinase, GSK3*β*. Constitutively active under basal conditions, GSK3*β* suppresses the anti-inflammatory factor IL-10.^22, 30, 34^ In line with these previous reports, our work suggests that RAW cell *Il6* and *Il10* expression is modulated by *p65*^+/−^-CM through an HGF/Met/GSK3*β* pathway.

During normal muscle repair, inflammatory macrophages transition to a proregenerative, or ‘M2' phenotype.^35^ This has been reported to involve AMP-activated protein kinase (AMPK).^36^ Recently, AMPK has been found to be negatively regulated by GSK3*β.*^37^ Interestingly, GSK3*β* activity has been reported to be elevated in a canine model of DMD, implicating it in muscular dystrophy.^38^ As we found that macrophages collected from injured *p65*^+/−^ muscle had higher percentages of cells bearing M2 markers (Supplementary Figure S2D), it is tempting to speculate that HGF/Met/GSK3*β* might be involved in macrophage phenotype switching and inflammation resolution in dystrophic muscle. It is worthy to consider however, that the mechanisms governing macrophage phenotype switching may differ between regeneration associated with acute *versus* chronic injuries. Future studies will be required to determine whether HGF/Met might have a role in this process.

The current DMD treatment, corticosteroid therapy, reduces inflammation, but comes with many unwanted side effects, including cataracts, growth impairment and reduction in bone density.^39^ An anti-NF-*κ*B therapy can only be temporary, at best, given the ubiquitous nature of this transcription factor, especially in immunity. Based on this investigation, the beneficial effect of anti-NF-*κ*B therapy might also be achieved by targeting HGF/Met. Phase II clinical trials using HGF mimetics for treating heart attack and delayed kidney graft function are currently in progress, suggesting that such an approach to DMD may well be feasible and safe.^40, 41^ Based on our findings, HGF/Met might be a new target for reducing inflammation, prolonging fiber integrity and delaying disease progression in dystrophic muscle.

## Materials and Methods

### Animals

C57Bl/6 (WT) mice and C57BL/10ScSn-*Dmd*^*mdx*^/J (*mdx*) mice were purchased from the Jackson laboratory (Bar Harbor, ME, USA). *P65*^+/−^ mice on a C57Bl/6 background, originally characterized by Beg *et al.*,^42^ were bred with *mdx* mice to produce *mdx*:*p65*^+/−^ and *mdx*:*p65*^*+/+*^ mice. P65 heterozygotes were backcrossed into an *mdx* background for a minimum of 10 generations. Genotyping was carried out by PCR analysis of tail samples. Mice ranged in age from 5 days to 12 weeks. Specific ages for each experiment are described below. All animal protocols used for these experiments were approved by the University of Pittsburgh's Institutional Animal Care and Use Committee.

### Cell culture

Primary WT and *p65*^+/−^ MDSCs and myoblasts (i.e. committed myogenic progenitor cells) were obtained from 5-month-old WT and *p65*^+/−^ mice using the modified preplate method, as described previously.^8^ MDSCs and myoblasts were cultured in proliferation medium (PM) containing 10% fetal bovine serum (FBS), 10% horse serum, 1% penicillin–streptomycin (P/S) and 0.5% chick embryo extract in DMEM. Freshly isolated myoblasts were minimally expanded *ex vivo* for 3 days and then treated with TNF*α* (10 ng/ml) for 0, 3, 6, 18, or 24 h. Cell pellets were immediately collected for RNA extraction. RAW264.7 cells, a murine macrophage-like cell line (ATCC, Manassas, VA, USA), were maintained and expanded in 10% FBS and 1% P/S in DMEM. Resident peritoneal macrophages were collected by lavage of the peritoneal cavity of unmanipulated mice and cultured in complete medium for 1 day before experiments. Primary macrophages were isolated from CTX-injured hindlimb muscles of WT (*n*=3) or *p65*^+/−^ (*n*=4) mice at 3 days postinjury, as reported previously.^43^ Cells were cultured *in vitro* for 3 days in DMEM supplemented with 10% FBS and 1% P/S on non-tissue culture-treated petri dishes. Primary macrophages were then removed from dishes by Versene solution for real-time RT-PCR or seeded for subsequent experiments. To block NF-*κ*B activation, WT MDSCs were treated with the reversible ATP-competitive inhibitor of IKK*β*, IKKi (EMD Millipore, Billerca, MA, USA) at 5 *μ*M. Recombinant mouse HGF was used at a concentration of 100 ng/ml (Pepro Tech, Rocky Hill, NJ, USA).

### Retroviral vector construction and transduction of MDSCs

To label the cells before *in vivo* and coculture experiments, MDSCs were retrovirally transduced to express nuclear-localized RFP. The retroviral vector was constructed with a combined CMV and long terminal repeat promoter driving RFP followed by a nuclear localization sequence derived from an SV40 large T-antigen. Briefly, cells were plated at 40% confluence and transduced at an MOI of 5 in culture medium supplemented with polybrene (8 μg/ml; Sigma-Aldrich, Milwaukee, WI, USA). After transduction, cells were passaged approximately four times to ensure stable gene expression. Finally, transduced WT and *p65*^+/−^ cells were selected by flow cytometry (FACSAria II, Bedford, MA, USA).

### Measurement of cell proliferation

In triplicate, cells were plated in a 24-well collagen type I-coated plate. Using a previously described live cell imaging (LCI) system, x10 brightfield images were taken in 10-min intervals over a 72- h period.^18^ Our custom-built LCI includes a biobox incubator that sits atop a Nikon Eclipse TE 2000 U microscope stage, which is attached to a CCD camera (Kairos Instruments LLC, Pittsburgh, PA, USA). Three locations to be imaged were randomly chosen per well, giving nine fields of view per population, per experiment. LCI was used to measure proliferation over 60 h by counting the number of cells per field of view at 12 -h intervals using ImageJ software (NIH, Bethesda, MD, USA). For coculture experiments, RFPn expressing WT or *p65*^+/−^ MDSCs were plated with murine RAW264.7 (ATCC) cells at a ratio of 1 : 10 and incubated overnight in PM. The following day, cells were activated by exposure to 100 ng/ml LPS (Sigma-Aldrich) in PM. Using LCI, we tracked the activity of RFP-expressing MDSCs over a 60- h period by capturing x10 brightfield and fluorescent images at 10-min intervals. PDT was calculated using a previously described model.^44^ For each field of view per population, the mean PDT was defined as the average of PDT measurements calculated at 48 and 60 h.

### CTX muscle injury model and stem cell implantation

WT (8–12 weeks old) mice were injured by the injection of 30 *μ*l of CTX (4*μ*M; Sigma) into the GAS muscle, as described previously.^45^ Twenty four hours later, 300 × 10^5^ RFPn-positive WT or *p65*^+/−^ MDSCs were injected into the injured GAS muscle. At 24 h, 72 h and 7 days postinjection, the animals were killed and the hind limbs were harvested and frozen in 2-methylbutane, and then precooled in liquid nitrogen. The specimens were stored at −80 °C until 10-*μ*m-thick cryosections were obtained at −25 °C. To examine muscle regeneration between genotypes, the GAS of 4- to 6-week-old *p65*^+/−^ and WT mice were injured with CTX, killed at 1, 3 or 5 days postinjury and tissues were harvested and snap frozen as described above.

### Immunofluorescence and histology

Cryosections were fixed with 5% formalin for 5 min and blocked with 10% donkey serum for 2 h. Slides were then incubated with one or more primary antibodies, including rabbit anti-RFP (1 : 200; Abcam, Cambridge, MA, USA), rabbit anti-mouse Ki-67 (1 : 200; Abcam), rabbit anti-phospho(S9)-GSK3*β* (1 : 50; Abcam) or rat anti-CD68 (1 : 200; Abcam) in 10% donkey or goat serum. Next, sections were incubated with secondary antibodies including 594-conjugated anti-rabbit or anti-rat IgG (1 : 500; Invitrogen, Grand Island, NY, USA) and 488-conjugated anti-rabbit or anti-rat IgG (1 : 500; Invitrogen) in PBS for 30 min. We stained sections for eMyHC using a mouse anti-mouse eMyHC antibody (1 : 50; Developmental Studies Hybridoma Bank, University of Iowa, Iowa City, IA, USA) with a Mouse-on-Mouse (M.O.M.) Staining Kit (Vector Labs, Burlingame, CA, USA) according to the manufacturer's directions. To identify necrotic fibers, we used a biotinylated anti-mouse IgG antibody (1 : 300; Vector Labs) with the M.O.M. kit diluent, according to the manufacturer's directions. Alternatively, cryosections were fixed with 2% paraformaldehyde and stained with goat anti-mouse HGF primary antibody (15 *μ*g/ml; R&D Systems, Minneapolis, MN, USA) using the Cell & Tissue Staining HRP-DAB System (R&D Systems) according to the manufacturer's instructions. For immunocytochemistry, cultured cells were fixed with 4% paraformaldehyde for 5 min, blocked with 10% donkey serum for 1 h and then incubated overnight at 4 °C with one or more primary antibodies, including rat anti-mouse F4/80 (1 : 250; AbD Serotec, Kidlington, UK), rabbit anti-mouse RELM*α* (1 : 100; Thermo Scientific Pierce, Waltham, MA, USA) or rabbit anti-mouse CD163 (1 : 100, Biorbyt, Cambridge, UK), followed by incubation with secondary antibodies 594-conjugated anti-rat (1 : 500; Invitrogen) or 488-conjugated anti-rabbit (1 : 500; Invitrogen) for 1 h at room temperature. Nuclei were stained with DAPI (1 : 1000; Invitrogen) for 5 min at room temperature. Histological analysis was carried out by hematoxylin and eosin staining (H&E), as described previously.^46^

### Image acquisition and processing

Immunofluorescent or brightfield images were captured using an upright fluorescent microscope (Leica Microsystems Inc., Wetzlar, Germany) equipped with a digital Retiga camera (QImaging, Surrey, BC, Canada). Images were acquired using Northern Eclipse (Empix Imaging Inc., Cheektowaga, NY, USA) or QCapture (QImaging) and quantified using ImageJ software (NIH) or CellProfiler (Broad Institute, Cambridge, MA, USA). To analyze RFP, CD68, Ki-67 or eMyHC staining, x200 images were captured through the entire injury and engraftment area from the region of highest engraftment along the length of the muscle. To analyze CD68 and phospho(S9)-GSK3*β* staining, four x600 images were captured from the injured area of each muscle. To analyze IgG or CD68 staining on ZsGreen-transduced muscles, x200 fluorescent images were captured from three locations along the length of sections from the medial region of each muscle. The x100, x200 or x400 brightfield images were captured for H&E analysis, with the regions for imaging chosen in a similar manner as described above. Area was measured using Adobe Photoshop (Adobe Systems Inc., New York, NY, USA). Final images for figures were also prepared in Adobe Photoshop.

### *In vitro* inflammation assay

RAW264.7 cells were plated in a six-well plate with 10^5^ cells per well, and then incubated for 48 h to achieve high density. CM was prepared by plating 10^6^ MDSCs into a T-175 flask with 15 ml of medium. Following a 24 h incubation, the medium was collected and then filtered (0.22 *μ*m). RAW264.7 cultures were washed with PBS and then cultured in CM with or without 100 ng/ml LPS for 30 min, 3 h or 24 h, at which time cell lysates were collected. To block Met, cells were pretreated with SU11274 (EMD Millipore; 448101) in DMEM supplemented with 1% P/S for 2 h before exposure to CM (SF) also supplemented with SU11274.

### Western blot

Cell and tissue lysates were prepared in RIPA buffer (Sigma) supplemented with protease and phosphatase inhibitors (nos. 2 and 3, 1 : 100; Sigma) and quantified using the Bio-Rad Protein Assay (500-0001; Bio-Rad, Hercules, CA, USA). Immunoblotting was performed as described previously.^6^ Membranes were incubated with monoclonal antibodies (1 : 1000; Cell Signaling, Danvers, MA, USA) to pS9-GSK3*β*, total GSK3*β* or polyclonal rabbit anti-HGF (1 : 100; Santa Cruz, Dallas, TX, USA) at 4 °C overnight in 5% milk or BSA in TBST. Ponceau S (Sigma) staining or probing with an HRP-conjugated antibody to GAPDH (1 : 8000; Abcam) was used to evaluate loading. For detection of total or phospho(Y1234/1235)-Met (1 : 1000; Cell Signaling), cells were lysed in RIPA with the above inhibitors with the addition of 0.2% SDS. Membranes probed for total or phospho-Met were washed for 30 min in high salt buffer (TBST supplemented with 0.5 M NaCl and 0.2% SDS) before detection.

### Real-time RT-PCR

Total RNA was isolated using TRI Reagent (Sigma) and reverse transcribed using Maxima First-Strand cDNA Synthesis Kit (Thermo Scientific, Waltham, MA, USA) according to the manufacturer's protocols. Real-time PCR was carried out using the Maxima Syber Green Assay Kit (Thermo Scientific) with an iQ5 thermocycler (Bio-Rad), with *β*-actin serving as an endogenous control. Primers were designed using PRIMER-Blast (NCBI, Bethesda, MA, USA) and were as follows: *β-actin*, F: 5′-CCACACCCGCCACCAGTTCG-3′ and R: 5′-TACAGCCCGGGGAGCATCGT-3′; *Interleukin 6*, F: 5′-TCTGCAAGAGACTTCCATCCAGTTGC-3′ and R: 5′-AGCCTCCGACTTGTGAAGTGGT-3′; *Interleukin 1*, F: 5′-AAGCCTCGTGCTGTCGGACC-3′ and R: 5′-GCTTGGGATCCACACTCTCCAGC-3′; *Interleukin 10*, F: 5′-GCATGGCCCAGAAATCAAGG-3′ and R: 5′-AGGGGAGAAATCGATGACAGC-3′; *tumor necrosis factor α*, F: 5′-AGCCCACGTCGTAGCAAACCAC-3′ and R: 5′-CGGGGCAGCCTTGTCCCTTG-3′; *vascular endothelial growth factor*, F: 5′-GGCTTTACTGCTGTACCTCC-3′ and R: 5′-GCAGTAGCTTCGCTGGTAGA-3′; *transforming growth factor β*, F: 5′-CTAATGGTGGACCGCAACAAC-3′ and R: 5′-CACTGCTTCCCGAATGTCTGA-3′; *Inos*, F: 5′-GCTGCCTTCCTGCTGTCGCA-3′ and R: CCTGACCATCTCGGGTGCGG; *hepatocyte growth factor*, F: 5′-TCATATCTTCTGGGAGCCAGATGCT-3′ and R: 5′-GGTCCAAATTGACAATTGTAGGTGTAGT-3′.

### Enzyme-linked immunoassay

To detect IL-10 and HGF secretion by primary macrophages, freshly isolated cells were cultured *ex vivo* for a total of 5 days. Cells were then washed two times and maintained in DMEM with 1% P/S (SF) for additional 24 h. Supernatants were then collected and stored at −80 °C; cells were collected and counted. ELISA was performed to detect IL-10 and HGF using the Mouse IL-10 ELISA Kit (Abcam) and the Mouse/Rat HGF Quantikine ELISA Kit (R&D Systems), respectively. The concentration of IL-10 and HGF was normalized per 10^5^ cells.

### Construction of HGF shRNA, AAV vector production and AAV administration

We designed two HGF-shRNAs, each based on previously reported siRNA sequences.^47, 48^ We first tested the efficiency of HGF knockdown *in vitro* using C2C12 mouse myoblasts (ATCC). We chose the most efficient sequence (>60% reduction by western blot) for our continued experiments. Our shRNA targeted the sense sequence 5′-ACGAAGTCTGTGACATTCCTC-3′ (position in gene sequence: nucleotide 718–738) and antisense sequence 5′-GCGGAATGTCACAGACTTCGT-3′. The following oligos were synthesized by Invitrogen: GATC-sense-CTCGAG-antisense-TTTTTTT-G (forward) and AATTC-AAAAAAA-sense-CTCGAG-antisense-G (reverse). We chose to use AAV serotype 9 because of its unique tropism towards muscles, which results in high gene transfer efficiency.^25^ We designed an AAV construct containing a dual cassette consisting of the human U6 promoter driving HGF-shRNA, followed by a CMV promoter driving the ZsGreen reporter gene as described previously.^46^ An AAV vector with scrambled shRNA was designed as a control (ct-shRNA). At 5 days of age, we performed intraperitoneal injection with 100 *μ*l of virus titered at 5 × 10^12^ vg/ml. Four weeks later, animals were killed and the muscles and soft tissues were harvested. Each experimental and control group contained four to six mice.

### Statistics

Data are reported as mean±S.E.M. or mean±S.D., as indicated in figure legends. To compare two groups, a Student's *t*-test was used to determine significance. To compare three or more groups, we used a one-way ANOVA followed by Tukey's *post hoc* analysis. A *P*-value <0.05 was considered significant.

## Abbreviations

DMD: Duchenne muscular dystrophy
HGF: hepatocyte growth factor
MDSCs: muscle-derived stem cells
NF-*κ*B: nuclear factor-*κ*B
IKK*β*: I*κ*B kinase *β*
VEGF: vascular endothelial growth factor
WT: wild type
AAV: adeno-associated viral
CM: conditioned medium
Tnf*α*: tumor necrosis factor-*α*
Il1*β*: interleukin-6
Il6: interleukin-1*β*
SF: serum-free
RFP: red fluorescent protein
CTX: cardiotoxin
eMyHC: embryonic isoform of myosin heavy chain
PDT: population doubling time
Tgfb: transforming growth factor *β* 1
Inos: inducible nitric oxide synthase
IKKi: IKK-2 Inhibitor IV
GSK3*β*: glycogen synthase kinase 3*β*
RELM *α*: resistin-like molecule *α*
GAS: gastrocnemius
DIA: diaphragm
AMPK: AMP-activated protein kinase
FBS: fetal bovine serum
HS: 10% horse serum
P/S: 1% penicillin–streptomycin
LCI: live cell imaging system
H&E: hematoxylin and eosin staining
HPF: high power field
ct-shRNA: control shRNA
