# Supplementary figures and images for: NF-κB inhibition reveals a novel role for HGF during skeletal muscle repair
Source: Cell Death Dis. 2015 Apr 23;6(4):e1730–. doi: 10.1038/cddis.2015.66 (PMC4650539; doi:10.1038/cddis.2015.66)

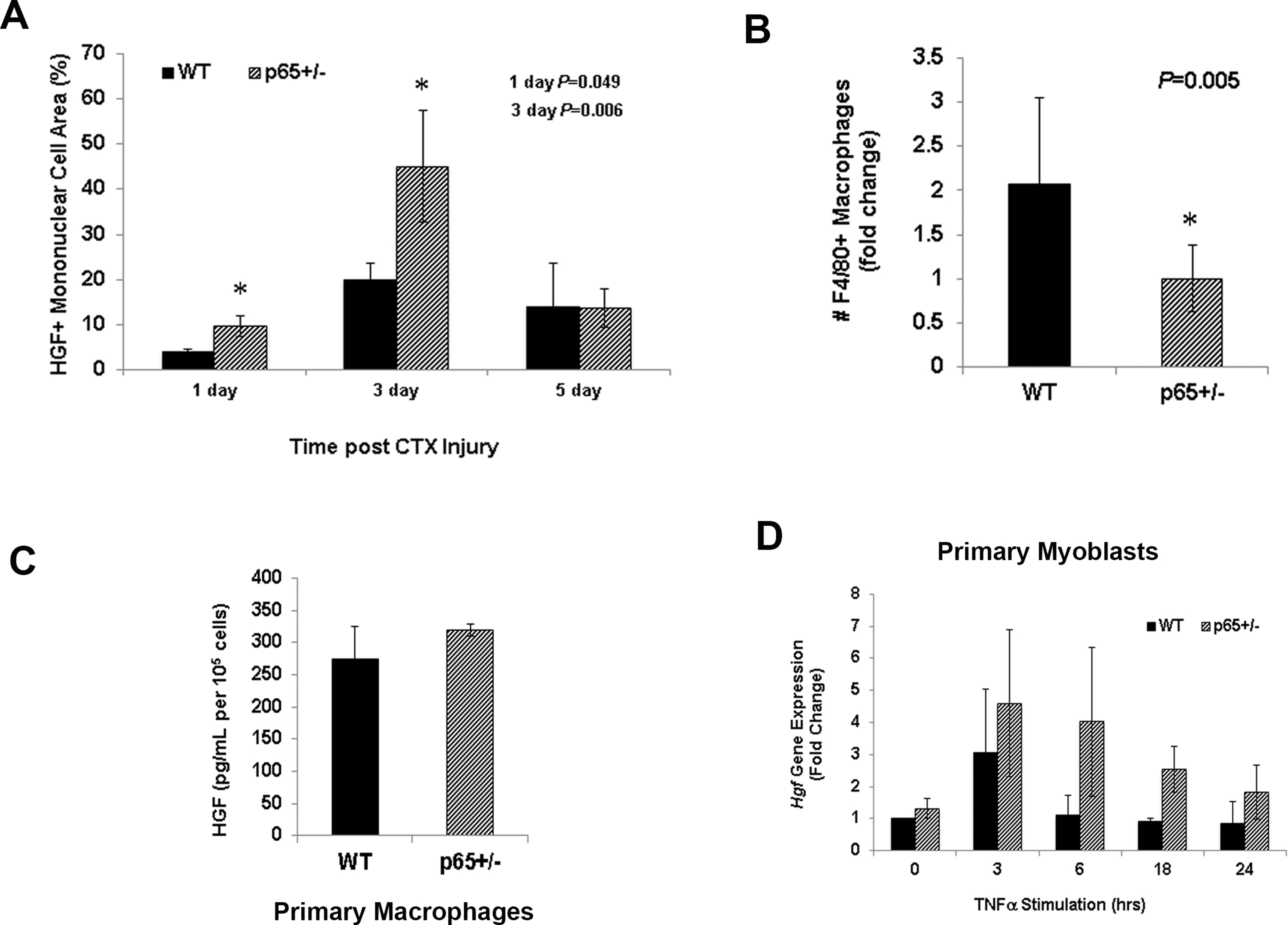

Supplement: Supplementary Figure 1 [file cddis201566x1.tif]

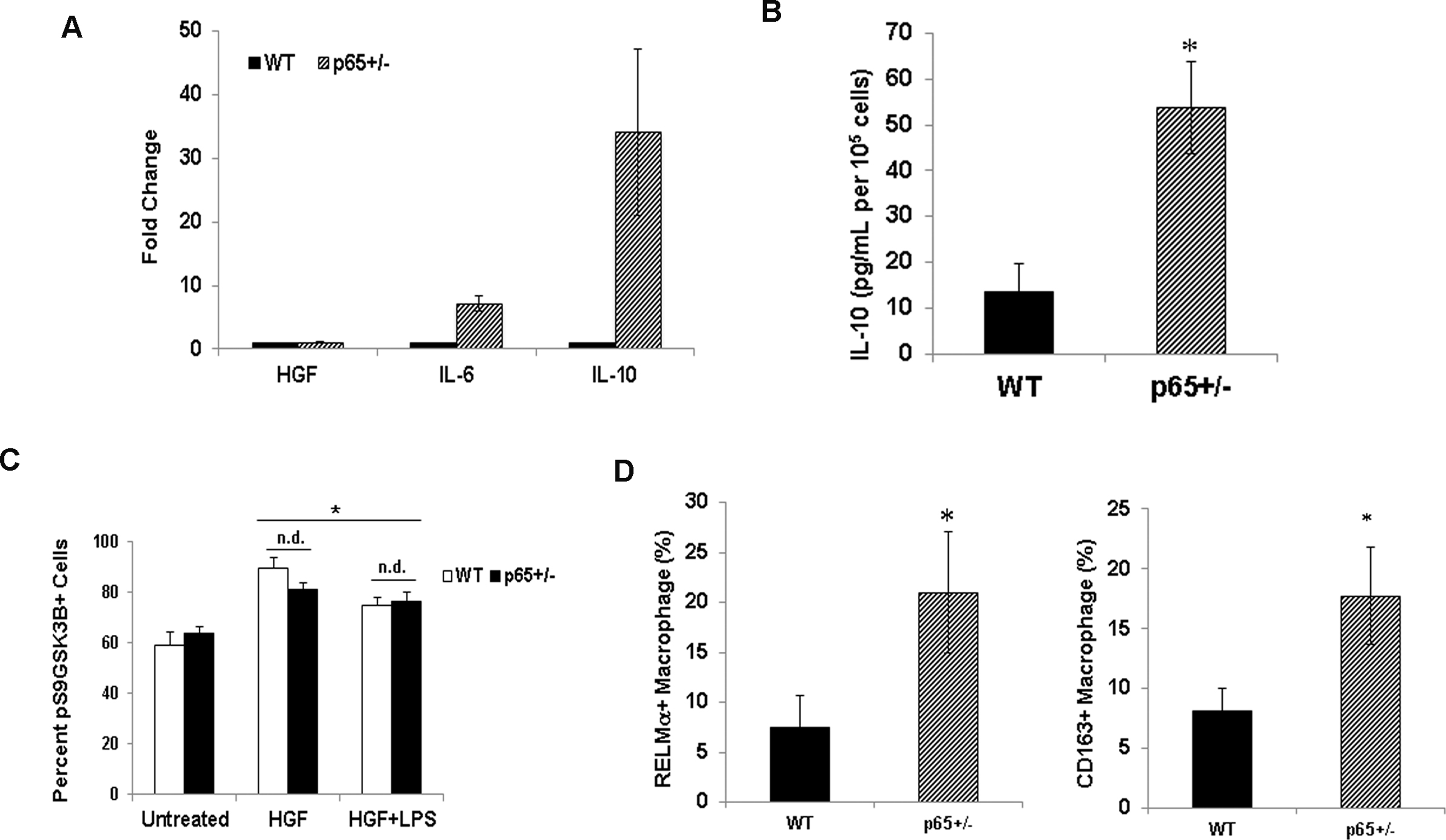

Supplement: Supplementary Figure 2 [file cddis201566x2.tif]

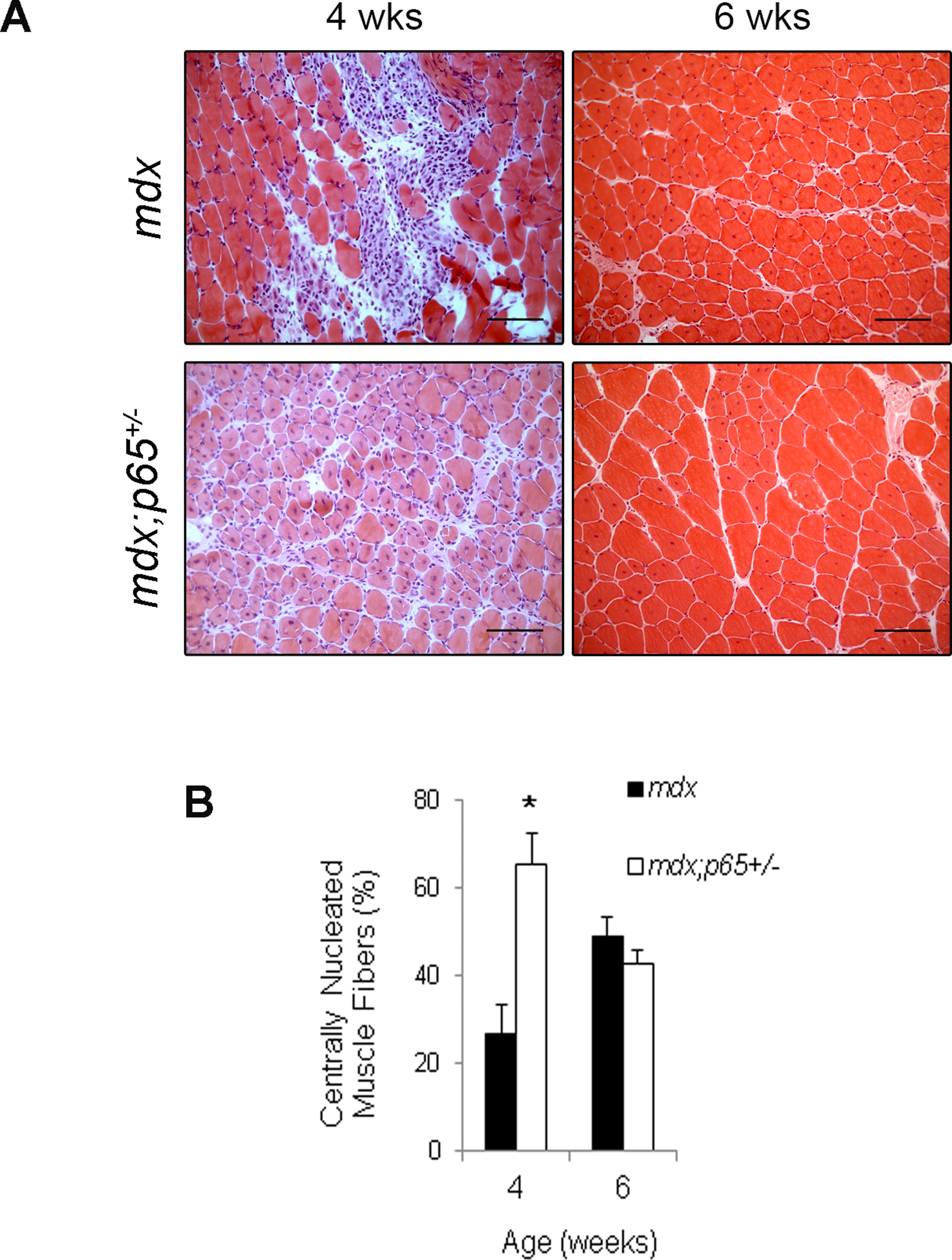

Supplement: Supplementary Figure 3 [file cddis201566x3.tif]

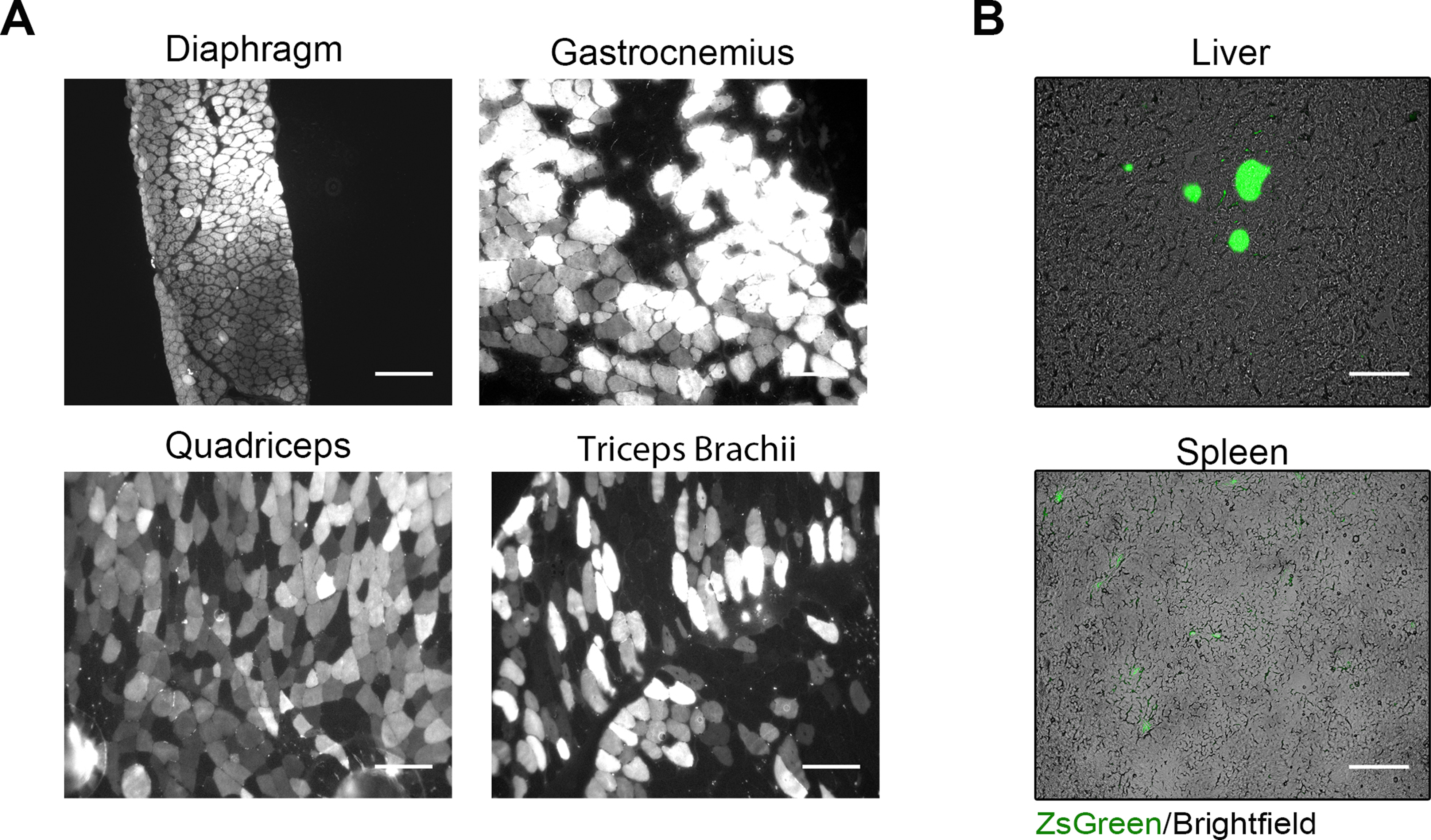

Supplement: Supplementary Figure 4 [file cddis201566x4.tif]
