# Supplementary material for: NF-κB inhibition reveals a novel role for HGF during skeletal muscle repair
Source: Cell Death Dis. 2015 Apr 23;6(4):e1730–. doi: 10.1038/cddis.2015.66 (PMC4650539; doi:10.1038/cddis.2015.66)
Supplement: Supplementary Figure Legends [file cddis201566x5.doc]

**Supplementary Figure 1.** (A) Three days after CTX injury of WT and *p65*^+/-^ gastrocnemius muscles, the area occupied by mononuclear cells expressing HGF was measured by immunohistochemical staining of tissue sections and normalized as percent of the total injured tissue area. At 1 day (*p*=0.049) and 3 days (*p*=0.006) post-injury, *p65*^+/-^ muscle had significantly more mononuclear HGF+ cells, suggesting more HGF+ cell infiltration, including both leukocytes and myogenic cell populations, during the regenerative phase of *p65*^+/-^ skeletal muscle (n=3 mice per group per time point). (B) Significantly less macrophages, identified as F4/80+ cells by immunofluorescent staining, were isolated from *p65*^+/-^ skeletal muscle at 3 days post-CTX injury compared to WT muscle (n=3-4). (C) Supernatants from macrophage cultures demonstrated that macrophages from *p65*^+/-^ muscle secreted only slightly more HGF (p=0.097) than cells from WT muscle. (D) However, TNFα stimulation induced greater *Hgf* expression in *p65*^+/-^ myoblasts compared to WT myoblasts. Data displayed as mean +/- standard deviation. Abbreviations: cardiotoxin, CTX; tumor necrosis factor α, TNFα.

**Supplementary Figure 2.** (A) Real time RT-PCR was performed to detect gene expression of *Hgf*, *Il6*, and *Il10* in macrophages collected from WT or *p65*^+/-^ muscle at day three post-injury and cultured *ex vivo*. We found that macrophages from injured *p65*^+/-^ and WT muscle expressed similar levels of *Hgf*; but those from *p65*^+/-^ muscle expressed 7-fold more *Il6* and 28-fold more *Il10* than WT counterparts. (B) ELISA of macrophage culture supernatants found that macrophages from *p65*^+/-^ muscle secreted significantly more IL-10 than macrophages collected from WT muscle (n=3-4, *p≤0.001). (C) WT and *p65*^+/-^ resident peritoneal macrophages do not demonstrate any differences in pS9-GSK3β upon treatment with HGF with or without LPS. (n=2, *p<0.05 vs Untreated). (D) Further analysis of macrophage phenotype using the M2 macrophage markers RELMα and CD163 revealed that macrophages from *p65*^+/-^ skeletal muscle had a significant increase in the percent of (left) RELMα+F4/80+ (p=0.003) and (right) CD163+F4/80+ (p=0.001) macrophages compared to macrophages collected from WT muscle, suggestive of an overall regenerative response in *p65*^+/-^ mice. Data displayed as mean +/- sem for C and mean +/- standard deviation for A, B, D. Abbreviations: resistin-like molecule α, RELMα.

**Supplementary Figure 3.** (A) Representative H&E staining of hind limb muscle from four week old *mdx* (top) and *mdx:p65^+/-^* (bottom) mice demonstrated that *p65* haploinsufficiency was associated with fiber regeneration during what is typically the degenerative phase of the dystrophic phenotype. By six weeks of age, larger fibers were evident in *mdx*:*p65^+/-^*  tissues. (B) Quantification of A. Scale bar: 100 µm. n=4-6 mice per group. *p<0.05.

**Supplementary Figure 4**. (A) I.p. injection of AAV vectors resulted body wide transduction of skeletal muscle, indicated by the ZsGreen reporter gene. Representative images of (B) livers (top) and spleens (bottom) from AAV-treated mice demonstrated minimal transduction.
